# Fungal nasal septum abscess caused by *Aspergillus flavus* complicating sinonasal surgery

**DOI:** 10.11604/pamj.2020.36.234.23186

**Published:** 2020-07-30

**Authors:** Youssef Rochdi, Youness Labani, Omar Oulghoul, Mohamed Mehdi El Fakiri, Hassan Nouri, Abdelaziz Raji

**Affiliations:** 1 ENT-HNS Department, Mohammed VI University Hospital Center, Marrakech, Morocco

**Keywords:** Fungal, nasal septum abscess, *Aspergillus flavus*, endoscopic surgery

## Abstract

The fungal nasal septum abscess is a rare localized invasive form of fungal rhinosinusitis. Rare cases have been described in the literature. In this article, we intend to describe a new case of fungal nasal septum abscess caused by Aspergillus flavus in diabetic patient after sinonasal surgery. A 53-year-old woman with a history of uncontrolled type 2 diabetes and asthma developed a nasal septum abscess after a sinonasal endoscopic surgery which was performed for nasal polyposis. Needle aspiration of the abscess was performed and the pus cultures were positive for Aspergillus flavus. The patient was treated with antifungal drugs and surgical drainage of the abscess. A clinical and biological improvement was observed. Her case has been followed up for 18 months, and there hasn't been any recurrence of the infection. The fungal nasal septum abscess should be suspected in patients who do not respond adequately to standard treatment of nasal septum abscess, especially patients with risk factors of fungal rhinosinusitis.

## Introduction

Fungal rhinosinusitis include fungal ball, allergic fungal rhinosinusitis and invasive fungal rhinosinusitis. The fungal nasal septum abscess is a rare localized invasive form. Rare cases have been described in the literature. Therefore, the aim this present paper is to report a new case of fungal nasal septum abscess caused by *Aspergillus flavus* in a diabetic patient after sinonasal surgery.

## Patient and observation

**Figure 1:**
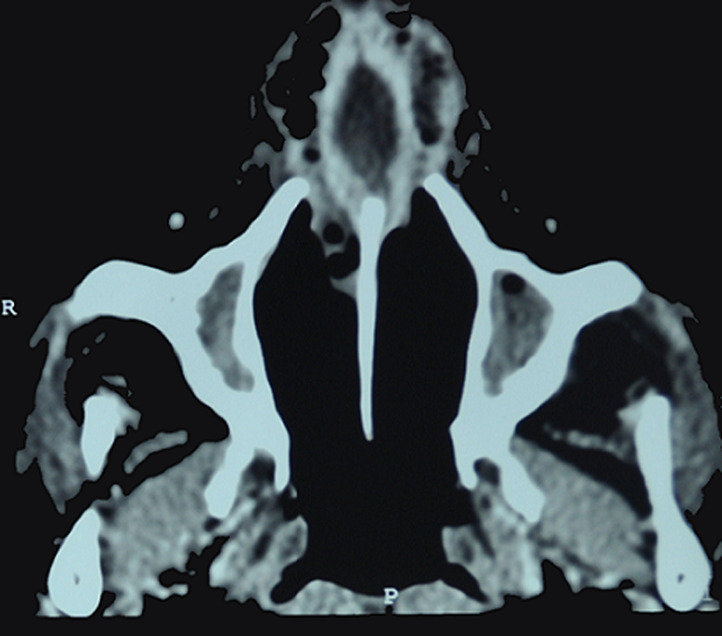
an axial computed tomography scan shows a collection in the anterior nasal septum

A 53-year-old woman with a history of uncontrolled type 2 diabetes and asthma developed a nasal septum abscess after a bilateral endoscopic ethmoidectomy performed for sinonasal polyposis. She was then treated with oral and nasal corticosteroids. Ten days after surgery, she developed a bilateral nasal obstruction with fever without any postoperative trauma. Upon physical examination, the patient was febrile with a temperature of 37.8°C. Anterior rhinoscopy showed a bulging anterior nasal septum bilaterally. The rest of physical examination presented no evidence of orbital, neurological, oral or facial involvement. Laboratory tests showed leucocytosis at 1520 cells/mm^3^with neutrophilic predominance at 1010 cells/mm^3^and a C-reactive protein (CRP) level of 100mg/l. The computed tomography (CT) scan revealed a fluid collection with thin rim enhancement in the anterior nasal septum without complications such as intracranial, facial or orbital extensions (Figure 1). Pus from the nasal septum was collected by needle aspiration, and sent to microbiology culture. Empirical antimicrobial therapy with intravenous amoxicillin/clavulanic acid and gentamicin was initiated. Final cultures were positive for *Aspergillus flavus* and no bacteria were detected. Pus from the nasal septum was cultured twice and the results were similar in both cultures. The patient was tested human immunodeficiency virus (HIV) negative. Operative drainage of nasal septum abscess was performed under general anesthesia by incision, debridement and nasal packing. The chosen therapy was oral voriconazole 200mg twice a day for one week followed by fluconazole 150 mg daily for one month. Insulin therapy was started for improvement and maintenance of glycemic control. On the one hand, clinical and biological improvement was achieved with the disappearance of clinical signs and normalization of the complete blood count and the CRP, on the other hand, the patient developed a saddle nose deformity one month after surgery. Her case has been followed up for 18 months and there has not been any recurrence of the infection.

## Discussion

The fungal nasal septum abscess is a very rare clinical manifestation. Two cases of fungal nasal septum abscess caused by *Aspergillus flavus* have been reported in the literature. The patients were a 64-year-old man with a history of crohn´s disease and pulmonary fibrosis treated with immunosuppressive medications and a 17-year-old adolescent boy with a history of T cell lymphoblastic lymphoma treated by chemotherapy. The fungal abscess occurred spontaneously in both cases without any trauma and the pathogens were isolated from culture. Both patients underwent antifungal therapy with voriconazole and surgical drainage of abscess. There was no recurrence or complication on the follow up [1,2]. Two cases of fungal nasal septum abscess caused by *Scedosporium apiospermum* have been reported. The first case was a 51-year-old woman with a history of asthma and recent treatment with oral steroids who presented a fungal septum abscess after endoscopic surgery performed for bilateral maxillary sinus fungus balls [3]. The second case was a 59-year-old man with uncontrolled diabetes who developed a fungal nasal septum abscess with a complication of orbital apex syndrome [4]. Two cases of fungal nasal septum abscess caused by atypical fungal stains such as *Fusarium verticillioides* and *Aspergillus thermomutatus* were reported in hematopoietic stem cell transplant recipients 5,6]. Our patient presented a fungal nasal septum abscess caused by *Aspergillus flavus* which was isolated from pus culture. The predisposing factors identified in this case were immune dysfunction with diabetes mellitus, nasal mucosal injury after endoscopic sinonasal surgery and the use of oral and nasal corticosteroids.

Review of the published cases showed similar clinical presentations between fungal and bacterial nasal septum abscess. The fungal abscess should be suspected in patients who do respond adequately to initial treatment with antibiotics and pus drainage, in immunocompromised patients treated by chemotherapy or immunosuppressive medications and in patients who have undergone endoscopic surgery after fungal rhinosinusitis. The diagnosis of fungal rhinosinusitis is based on microbiological and histopathological methods. Fungal culture is the gold standard method for diagnosis [7]. The management of fungal nasal septum abscess is based on medical therapy with antifungal drugs and surgical drainage and debridement. Voriconazole is recommended for the primary treatment of invasive sinonasal aspergillosis. It´s initiated with a loading dose of 6 mg/kg IV every 12 h for 2 doses, followed by 4 mg/kg every 12 h. Duration of antifungal therapy is continued for a minimum of 6-12 weeks; in immunosuppressed patients, therapy should be continued throughout the period of immunosuppression and until lesions have resolved [8]. In our case report, we treated our patient by oral voriconazole 200mg twice daily for a week due to the unavailability of the injection form; and we followed the treatment by fluconazole 150 mg daily for a month because of the high cost of voriconazole and the initial improvement after treatment. Early diagnosis and optimal treatment reduce disease progression and the risk of complications. Nonetheless, once it is not treated properly, life threatening complications may occur specially in immunocompromised patients such as those involving the central nervous system.

## Conclusion

The fungal nasal septum abscess is a rare condition. It should be suspected in patients with nasal septum abscess and inadequate response to standard treatment especially in patients with risk factors of fungal rhinosinusitis. Early diagnosis and optimal treatment prevent serious complications.
